# Editorial: The plant cell wall: advances and current perspectives

**DOI:** 10.3389/fpls.2023.1235749

**Published:** 2023-06-22

**Authors:** Wagner Rodrigo de Souza, Rowan A.C. Mitchell, Igor Cesarino

**Affiliations:** ^1^ Center for Natural and Human Sciences, Federal University of ABC, Santo André, Brazil; ^2^ Plant Sciences, Rothamsted Research, Hertfordshire, United Kingdom; ^3^ Departamento de Botânica, Instituto de Biociências, Universidade de São Paulo, São Paulo, Brazil; ^4^ Synthetic and Systems Biology Center, InovaUSP, São Paulo, Brazil

**Keywords:** plant cell wall, biomass, cellulose, hemicellulose, lignin, bioeconomy

Plant cells are surrounded by the cell wall, a dynamic component that shapes the cell and is key to their function. In developing tissues with an active cell elongation process, plant cells are surrounded by a primary cell wall (PCW), comprised of cellulose, hemicellulose, pectins, structural proteins, and, in grasses, phenolic compounds (Loqué et al., 2015). The PCW is responsible for maintaining cell shape and expanding cell size, bestowing it with mechanical strength, minimizing water loss and protecting it against stresses. A secondary cell wall (SCW) is deposited internally to the PCW once cell elongation ceases to allow specialized cells to perform their function. SCWs are composed of cellulose and hemicellulose impregnated by the phenolic polymer lignin, resulting in a complex and rigid structure that provides physical strength and hydrophobicity to supportive and water-transporting tissues (Meents et al., 2018). Cell walls also constitute the majority of plant biomass and thus play a crucial role in the food and biofuel industries (Burton and Fincher, 2014). Given that PCWs are thought to contain just a few layers of cellulose microfibrils whereas the SCWs contain hundreds, lignified SCWs account for the majority of plant biomass (Zeng et al., 2014). The proportions and chemical composition of the major components of the SCW also vary among cell types and plant species, and their physicochemical properties will ultimately determine biomass digestibility, thereby modulating nutrient release, gut biota, and health (Zhao et al., 2012; Burton and Fincher, 2014). This property also influences the production of biofuels, e.g. biomass fermentation to produce bioethanol. Understanding the distinct aspects of cell wall biology is therefore crucial for improving the production of plant-based biomaterials and developing plants with important characteristics for the food, agricultural, and bioenergy industries.

This Research Topic aimed to collate a wide spectrum of perspectives and advances in plant cell wall research. Thirteen articles were accepted for publication, and they are organized into two sections: 1) Advances in plant cell wall deposition/assembly/biogenesis; and 2) Biotechnological strategies toward optimized plant cell walls for the bioeconomy.

## Advances in plant cell wall deposition/assembly/biogenesis

In this Research Topic, important advances in plant cell wall biogenesis were reported. Paterlini et al. performed biochemical characterization of the cell wall of *Arabidopsis thaliana* plasmodesmata, which are membrane-lined pores involved in the symplastic transport of biological molecules between neighbouring cells. Xyloglucans and pectins were shown to account for around 60% of the plasmodesmata cell wall, whereas enzymatic fingerprinting revealed specific polysaccharide signatures: most xyloglucans were fucosylated, homogalacturonans were not extensively methyl-esterified, rhamnogalacturonan I showed limited branching and rhamnogalacturonan II was highly methyl-acetylated. These data open new opportunities for the study of plasmodesmata function. Li et al. found numerous cell wall-related transcripts were differentially regulated when comparing transcriptomes during endosperm cellularization and endosperm differentiation of Arabidopsis wild-type and the *N-terminal acetyltransferase A subunit 15* (*naa15*) mutant where these processes are abnormal. Chen et al. used a rice rolling-leaf mutant, *rlm1-D*, to demonstrate that its phenotype, characterized by rolling leaves, is mainly caused by abnormal secondary cell wall (SCW) deposition. *RLM1* was cloned by a map-based method and found to encode an R2R3 MYB transcription factor that can bind to the promoter of *CINNAMYL ALCOHOL DEHYDROGENASE 2* (*OsCAD2*), a key gene responsible for lignin biosynthesis in rice. An interacting partner of RLM1, MITOGEN-ACTIVATED PROTEIN KINASE 10 (OsMAPK10), was also identified and the authors proposed a MAPK-MYB-*OsCAD2* genetic regulatory network controlling SCW deposition, providing novel insights into the molecular regulatory mechanisms controlling leaf morphology in rice.

By analyzing published pressure–volume curves and measures of succulence in 25 species of the order Caryophyllales, Fradera-Soler et al. showed that elastic adjustment, whereby plants change cell wall elasticity, is uniquely beneficial to succulents for avoiding turgor loss. The authors also studied phylogenetically diverse succulent species to demonstrate several differences in cell wall biochemistry between succulent and non-succulent leaves, pointing to the existence of what they called “succulent glycome”. Dabravolski and Isayenkov discuss in a review the roles of cell wall components in salt stress tolerance and the regulatory mechanisms underlying cell wall maintenance under salt stress conditions. Xylan is emerging as the key polymer in SCW for linking polysaccharide and lignin components but is also present in PCW (Terrett and Dupree, 2019; Tryfona et al., 2023). New results suggesting separate xylan synthase complexes for PCW and SCW in Arabidopsis are discussed by Anders et al. In grass PCW and SCW, xylan is decorated with arabinose some of which have hydroxycinnamates attached (Scheller and Ulvskov, 2010; Terrett and Dupree, 2019). There are two articles on AT10, the BAHD acyltransferase enzyme responsible for addition of *p*-coumarate to arabinoxylan. Moller et al. showed that abolition of *OsAT10* by CRISPR/Cas9 almost abolished this linkage in rice. Houston et al. reported that a natural knock-out variant of the *HvAT10* ortholog in barley caused much lower levels of this linkage in barley grain cell walls. The role of *p*-coumarate on arabinoxylan is unclear but the similar ferulate decoration appears to be crucial in cross-linking arabinoxylan chains to each other and to lignin in grass cell walls as discussed in the review article by Chandrakanth et al. which also covers hydroxycinnamate decoration of lignin. Another feature of grass cell walls is the presence of (1,3;1,4)- β-glucan synthesized by CELLULOSE SYNTHASE-LIKE F6 (CSLF6). New evidence on control of transcription of this gene in barley grain is presented in Garcia-Gimenez et al.

## Biotechnological strategies toward optimized plant cell walls for the bioeconomy

Engineering cell walls is a key strategy to generate optimized crops with enhanced processability to produce biofuels and other bioproducts in biorefineries (Loqué et al., 2015). When targeting lignin, two strategies are envisaged: reducing lignin content and altering lignin structure/composition. De Meester et al. reported on the engineering of curcumin, a natural metabolite harboring two phenolic rings linked by a labile aliphatic chain, as an alternative monomer incorporating into poplar lignin. By expressing two curcumin biosynthetic genes under the control of a SCW-specific promoter, curcumin was produced and incorporated into the lignified cell walls of poplar. However, different from what has been reported for Arabidopsis (Oyarce et al., 2019), the curcumin-producing transgenic poplars suffered from yield penalties in addition to altered cell wall composition. More importantly, the saccharification efficiency of the transgenic lines was not different from that of the control plants, suggesting that translating this strategy from Arabidopsis to crops will likely demand further optimization. In another strategy to alter lignin composition, Shafiei et al. reported on the down-regulation of the lignin biosynthetic gene *ferulate 5-hydroxylase* (*F5H*) in barley, which resulted in reduced syringyl/guaiacyl (S/G) ratio in the straw. Interestingly, parameters such as lignin content, straw mechanical properties, plant growth habit, grain characteristics, and saccharification efficiency all remained unaffected. These results suggest that altering S/G composition had little effect on plant development and biomass processability in barley. For grass biomass in general, targeting the ferulate responsible for cross-linking lignin to polysaccharide in SCW is seen as a promising approach to enhance saccharification, as is boosting of ester-linked ferulate on lignin in all plant biomass, both achieved by manipulation of *BAHD* genes (Chandrakanth et al.). Bioenergy and biorefining require specific biomass characteristics of a crop variety. Based on a detailed cell wall analyses of above-ground biomass (comprised of stem and leaf material) of 49 representative genotypes of the genus Miscanthus, Iacono et al. identified a number of cell wall related variables important for biomass recalcitrance. Their results emphasize the inter- and intra- specific variation in cell wall characteristics and biomass recalcitrance and the importance of also considering yield- and organ-related parameters when analyzing cell wall properties and biomass recalcitrance aimed at improving Miscanthus as a biomass crop.

The characterization of the molecular mechanisms underlying the biosynthesis and deposition of the major cell wall components is essential not only for our understanding of how cell walls evolved as a dynamic component playing key roles in plant growth and development but also to allow the rational engineering of plant cell walls for the bioeconomy. Articles in this Research Topic provide novel insights into cell wall biogenesis and exciting biotechnological strategies for the optimization of plant biomass for biorefineries.

## Author contributions

WS, IC and RM wrote about every article they each edited. All authors provided feedback on the Editorial. All authors contributed to the article and approved the submitted version.
